# Hardness Prediction of Grind-Hardening Layer Based on Integrated Approach of Finite Element and Cellular Automata

**DOI:** 10.3390/ma14195651

**Published:** 2021-09-28

**Authors:** Yu Guo, Minghe Liu, Yutao Yan

**Affiliations:** 1 School of Mechanical Engineering and Automation, Northeastern University, Shenyang 110819, China; guoyu@mail.neu.edu.cn (Y.G.); ytyan@mail.neu.edu.cn (Y.Y.); 2 School of Mechanical Engineering, Shenyang Jianzhu University, Shenyang 110168, China

**Keywords:** grind-hardening process, finite element, cellular automata, grinding temperature, microstructure transformation, hardness prediction

## Abstract

As an emerging composite processing technology, the grind-hardening process implements efficient removal on workpiece materials and surface strengthening by the effective utilization of grinding heat. The strengthening effect of grind-hardening on a workpiece surface is principally achieved by a hardened layer, which is chiefly composed of martensite. As a primary parameter to evaluate the strengthening effect, the hardness of the hardened layer mostly depends on the surface microstructure of the workpiece. On this basis, this paper integrated the finite element (FE) and cellular automata (CA) approach to explore the distribution and variation of the grinding temperature of the workpiece surface in a grind-hardening process. Moreover, the simulation of the transformation process of “initial microstructure–austenite–martensite” for the workpiece helps determine the martensite fraction and then predict the hardness of the hardened layer with different grinding parameters. Finally, the effectiveness of the hardness prediction is confirmed by the grind-hardening experiment. Both the theoretical analysis and experiment results show that the variation in the grinding temperature will cause the formation to a certain depth of a hardened layer on the workpiece surface in the grind-hardening process. Actually, the martensite fraction determines the hardness of the hardened layer. As the grinding depth and feeding speed increase, the martensite fraction grows, which results in an increase in its hardness value.

## 1. Introduction

As a traditional machining process, grinding technology, which is usually applied in the final processing, can not only achieve higher machining accuracy but also guarantee the requirements of the surface integrity (especially surface roughness) of the part [1,2], whereas with the increasing requirements of modern industry on the application performance of parts, the traditional grinding process has gradually been unable to meet the demand of industrial production. Therefore, the development of a new grinding technology would attract a lot of attention from scholars in the field of machinery manufacturing nowadays.

Generally, grind-hardening is defined as an integrated process that simultaneously integrates efficient material removal and the surface strengthening of the workpiece [3]. Unlike the traditional process, grind-hardening can not only achieve efficient material removal at a greater grinding depth but can also form a hardened layer dominated by martensite on the workpiece surface, which effectively makes use of the grinding heat. The formed hardened layer achieves the effect of strengthening on the surface of the part. Moreover, in order to achieve the right temperature for microstructure transformation on the workpiece surface, the grind-hardening process usually adopts dry grinding, namely without the participation of a coolant, which prevents environmental pollution and thereby realizes green manufacturing [4]. Currently, there have been a lot of related studies on the formation mechanism of the hardened layer and the influence of the workpiece material, grinding parameters, temperature, and cooling modes on the hardened layer. Nguyen et al. [5] presented a temperature-dependent finite element (FE) heat transfer model, which analyzed the influence of grinding temperature distribution and different cooling modes on the formation of the hardened layer. Alonso et al. [6] proposed a method for controlling the hardness penetration depth by changing the grinding parameters with the help of a cylindrical grind-hardening experiment and metallographic analysis. Salonitis et al. [7] analyzed the influence mechanism of the grinding temperature on the formation of a hardened layer with FE method and predicted the residual stress. Zhang et al. [8] performed an FE simulation and grind-hardening experiment to explore the influence of different grinding parameters on the depth, hardness, and consistency of the hardened layer for 45 steel and 40Cr steel. Huang et al. [9,10] conducted a grind-hardening experiment on AISI 5140 steel for analyzing the formation mechanism of a hardened layer with the influence of a minimum quantity of lubrication and predicting its surface roughness.

From the literature outlined above, it can be seen that the hardened layer is mainly produced by microstructure transformation on the workpiece surface with the grinding heat effect. Meanwhile, the hardened layer with a different microstructure determines the strengthening effect of the workpiece surface. In current studies, the FE method is commonly applied to explore the influence of the grinding heat on the formation of a hardened layer during the grind-hardening process. However, the microstructure transformation process of the hardened layer is always ignored. As an adaptive approach for the microstructure transformation simulation of metallic materials, cellular automata (CA) has a distinctive characteristic of comprehensively and intuitively reflecting the microstructure transformation process of metal materials under different processing conditions. Meanwhile, the approach is capable of accurately calculating the grain size, fraction and distribution of the final microstructure [11]. Lan et al. [12] simulated the transformation from austenite to ferrite for low-carbon steel in continuous cooling, and predicted the average ferrite size at a different cooling rate with a two-dimensional CA approach. Bos et al. [13] established a CA model to simulate the microstructure recrystallization and transformation of the dual-phase steel during the annealing process. Zhi et al. [14] conducted a CA simulation on the martensite transformation of the high strength and elongation steel in the quenching process and analyzed the martensite fraction at different cooling temperatures. Shabaniverki et al. [15] proposed a model to assess the softening kinetics and simulated the microstructure transformation during isothermal annealing within the aluminum alloy by the CA approach. From the above studies, the CA approach has been widely used to simulate the transformation of metallic materials. Therefore, it is known that the CA approach is capable of simulating the microstructure transformation, which is helpful to study the formation of hardened layer in grind-hardening process.

As an important parameter to characterize material properties, hardness affects the macro-mechanical properties and application performances of parts. Moreover, the hardness of the hardened layer, whose value mainly depends on the surface microstructure, is also a key to evaluate the strengthening effect on the surface. From this consideration, the integration of FE and CA approaches is helpful to discover the distribution and variation of the grinding temperature of the workpiece and simulate microstructure transformation. Furthermore, the hardness superposition principle is applied to predict the hardness of the hardened layer with different grinding parameters. Eventually, the effectiveness of the hardness prediction was verified by a grind-hardening experiment. The research may contribute to comprehensively reveal the formation mechanism of the hardened layer and improve the evaluation system of grind-hardening technology on the strengthening effect of the workpiece surface. This study is of great scientific significance and application value.

## 2. Heat Source Modeling and FE Simulation

### 2.1. Heat Source Model

According to the literature review, the formation of the hardened layer in the grind-hardening process mainly depends on improving the grinding temperature of the workpiece surface by controlling the grinding depth and feeding speed, thereby transforming the microstructure into martensite. Therefore, the analysis of the temperature distribution and its variation are the prerequisite for studying the microstructure transformation and achieving the hardness prediction of the hardened layer.

During the grinding process, the material removal by the abrasive grains has experienced three stages, namely sliding, ploughing, and cutting. Among them, sliding and ploughing play key roles in the generation of grinding heat. Since distribution and grain size on the wheel are random, a large number of small grains merely have the function of sliding and ploughing without generating chips. This causes a large amount of grinding heat generated during the grinding process. The heat is usually expressed by the total heat flux *q*_t_, which is a function related to the tangential grinding force, wheel characteristics, and grinding parameters [16,17], namely:

(1) $$q_{t}=\frac{F_{t}\times v_{s}}{b\times l},$$

where *F*_t_ is the tangential grinding force; *v*_s_ is the wheel speed; *b* is the width of the wheel; *l* is the contact arc length between the wheel and the workpiece; and $l=\sqrt{a_{e}\times d_{\mathrm{se}}}$; *a*_e_ is the grinding depth; and *d*_se_ is the wheel diameter.

*F*_t_ can be expressed as

(2) $$F_{t}=0.4\times 28,282\times {\left(a_{e}\right)}^{0.86}\times {\left(v_{s}\right)}^{-1.06}\times {\left(60v_{w}\right)}^{0.44},$$

where *v*_w_ is the feeding speed.

**Figure 1:**
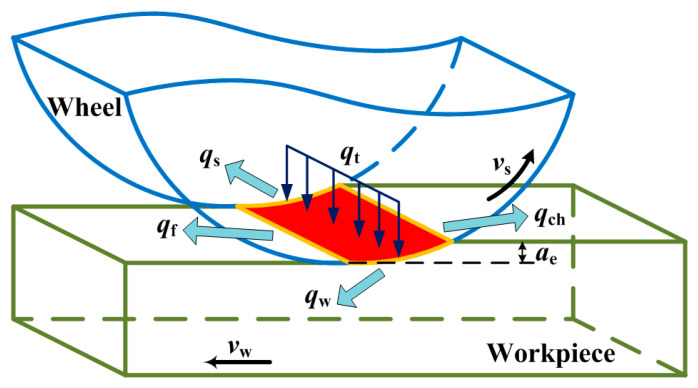
Schematic diagram of grinding heat transfer.

Once the grinding heat is generated, it will transfer to the workpiece, chips, wheel, and coolant via heat conduction and heat convection, as shown in Figure 1.

Therefore, the distribution model of total heat flux can be given by [18]

(3) $$q_{t}=q_{w}+q_{\mathrm{ch}}+q_{s}+q_{f},$$

where *q*_w_, *q*_ch_, *q*_s_, and *q*_f_ are the heat flux density transferred into the workpiece, chips, wheel, and coolant, respectively.

Among others, *q*_ch_ is given by [19]

(4) $$q_{\mathrm{ch}}=\rho_{w}\times C_{w}\times T_{\mathrm{mp}}\times \frac{a_{e}\times v_{w}}{l},$$

where *ρ*_w_, *C*_w_, and *T*_mp_ are the density, specific heat, and melting temperature of the workpiece material, respectively.

Usually, dry grinding is adopted in the grind-hardening process to ensure that the surface of the workpiece reaches a high temperature. Therefore, it is considered that no heat flux is transferred to the coolant, namely *q*_f_ = 0. *q*_w_ can be expressed as

(5) $$q_{w}=R_{w}\times \left(q_{t}-q_{\mathrm{ch}}\right),$$

where *R*_w_ is the heat partition ratio transferred to the workpiece, which is given by

(6) $$R_{w}=\frac{1}{\sqrt{1+\frac{\lambda_{s}\times \rho_{s}\times C_{s}\times v_{s}}{\lambda_{w}\times \rho_{w}\times C_{w}\times v_{w}}}},$$

where *λ*_s_, *ρ*_s_, and *C*_s_ are the heat conductivity, density, and specific heat of the wheel material, respectively; *λ*_w_ is the thermal conductivity of the workpiece material.

### 2.2. FE Simulation of Temperature Field

**Figure 2:**
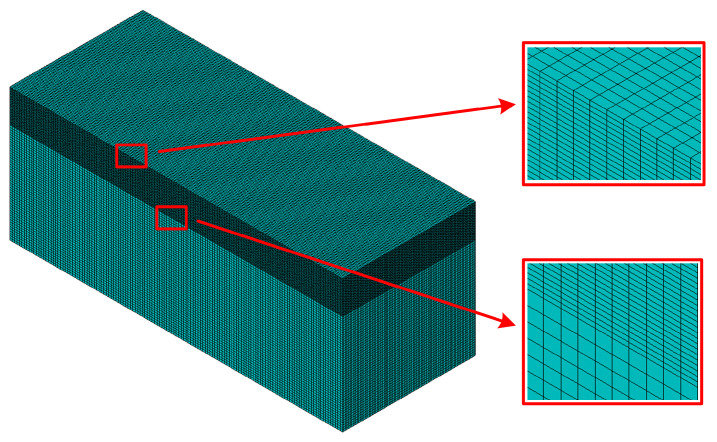
Mesh division of workpiece.

The FE method was applied to simulate the temperature field of the workpiece in the grinding process. In the simulation, the workpiece material is 1045 steel, whose chemical composition is shown in Table 1, and the material properties are listed in Table 2. The size of the workpiece is 90 mm × 9 mm × 14 mm. Since this study mainly focus on the distribution and variation of the grinding temperature on the workpiece, the FE model of the wheel is not set up in the FE simulation. The grind-hardening process only produces a strengthening effect on the workpiece surface. It is necessary to refine the finite element size on the workpiece surface to make the simulation result more accurate during the mesh division, as shown in Figure 2. In Figure 2, the finite element size for the workpiece surface is 0.45 mm × 0.09 mm × 0.04 mm and the finite element size for the matrix is 0.45 mm × 0.09 mm × 0.24 mm. Linear heat source model is adopted in the study, as shown in Figure 1. Taking into account the fact that the wheel is moving along the feeding direction in the cutting area, it is necessary to ensure that the grinding heat source moves at a certain feeding speed during the simulation.

**Figure 3:**
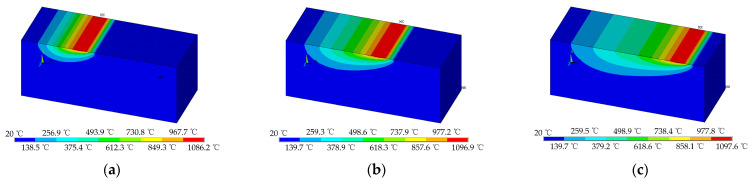
Grinding temperature distribution at different times (*a*_e_ = 350 μm, *v*_w_ = 9 m/min, *v*_s_= 26.4 m/s): (**a**) 0.15 s; (**b**) 0.3 s; and (**c**) 0.45 s.

**Figure 4:**
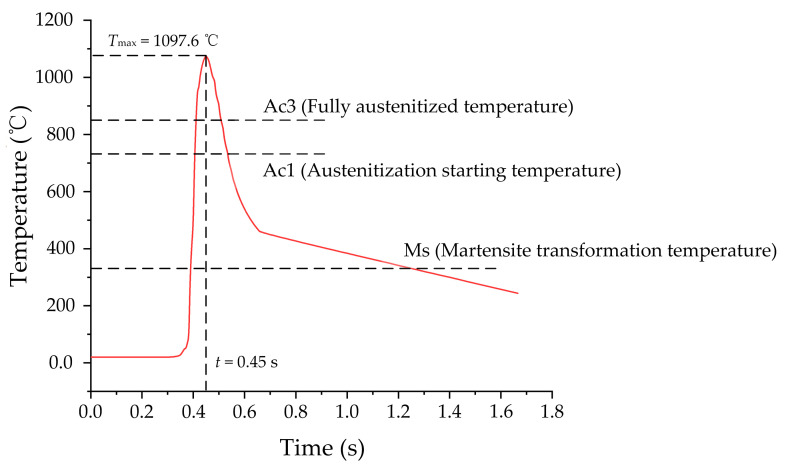
The instantaneous variation of grinding temperature.

Figure 3 shows the grinding temperature distribution of the workpiece surface at different times (*t* = 0.15, 0.3 and 0.45 s). It can be seen from Figure 3 that the grinding temperature varies along the feeding direction, which is in line with the actual process of the temperature variation when the wheel cuts the workpiece. Additionally, a quite high temperature appears on the surface, which can reach a maximum of 1097.6 °C. Due to the effect of heat conduction inside the workpiece, there is a certain gradient in the grinding temperature at different positions along the depth of the workpiece. The instantaneous temperature variation on the workpiece surface is shown in Figure 4.

The grinding temperature presents a trend of first rising and then decreasing, as shown in Figure 4. Due to the cutting action of the wheel on the workpiece, the grinding temperature rises rapidly to the maximum which is more than fully austenitized temperature Ac3 when the wheel grinds to this area. According to the theory of phase transformation [20,21], the heating rate on the workpiece surface is so fast during the grind-hardening process that the initial microstructure starts to transform into austenite when the surface temperature of the 1045 steel workpiece exceeds the austenitization starting temperature Ac1. Then, the initial microstructure will be completely transformed into austenite when the grinding temperature reaches Ac3. Due to the relative movement between the wheel and workpiece, the heat conductivity inside the workpiece and the heat convection between the workpiece and the surrounding, the surface temperature of the workpiece will reach its highest temperature and then rapidly drop. Taking into account continuous cooling transformation (CCT) diagrams, it is known that the cooling rate of the workpiece surface is greater than the critical cooling rate of martensite, and the final temperature is lower than martensite transformation temperature Ms. Therefore, the austenite on the workpiece surface will transform into martensite during the cooling process. In summary, with the FE simulation of the grinding temperature, we can conclude that a hardened layer dominated by martensite was finally formed on the workpiece surface.

## 3. Simulation for Microstructure Transformation Based on CA Approach

The formation of a hardened layer for a 1045 steel workpiece usually experiences transformation from the initial microstructure first into austenite and then into martensite. Firstly, the austenitization process includes austenite nucleation, grain growth, and coarsening when the temperature is higher than Ac1. Then, the austenite transfers to martensite as the temperature drops lower than Ms. Therefore, kinetic models for different microstructure transformation processes and transformation rules need to be set up for simulating the formation of the hardened layer.

### 3.1. CA Modeling

**Figure 5:**
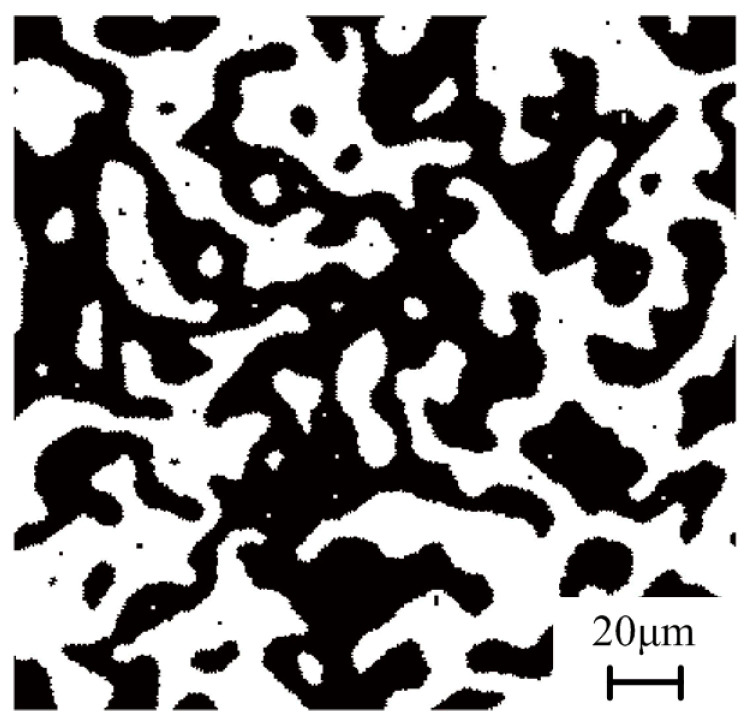
Initial microstructure distribution of 1045 steel workpiece.

**Figure 6:**
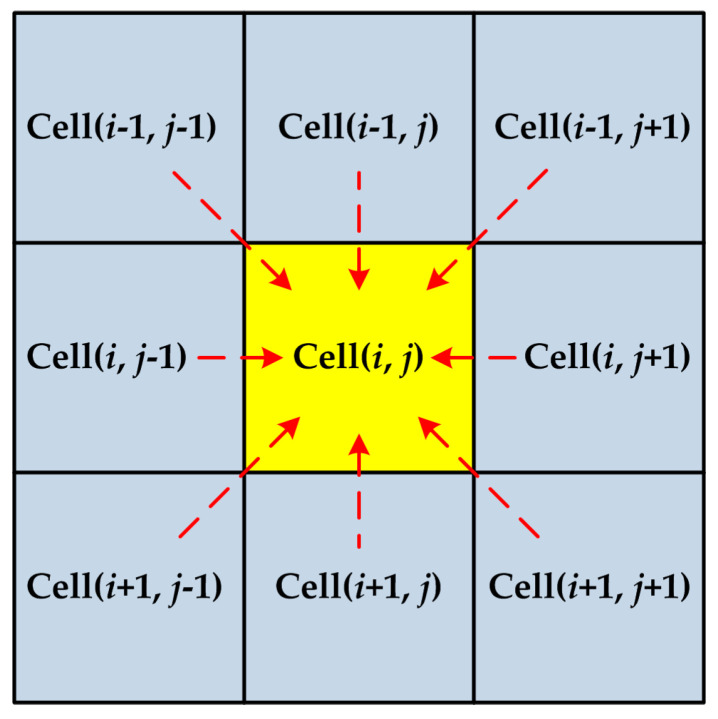
Moore neighbor in 2D Cellular Automata.

The CA approach is applied to simulate the microstructure transformation of the hardened layer for 1045 steel. The initial microstructure distribution of the simulated area on the surface is shown in Figure 5. In the figure, pearlite is colored in black, and ferrite is colored in white. The simulated area of the workpiece surface is equally divided into 400 × 400 square grids (namely cells), whose side length *L*_0_ = 1 μm; hence, the entire cell space is 400 × 400 μm^2^. As introduced in Figure 6, the Moore neighbor and periodic boundary condition were applied in this study. In addition, three state variables are assigned to each cell—(1) Crystallographic orientation variable: pearlite is set to 0 and ferrite is set to 200; the austenite with different crystallographic orientation is set to a random integer from 11 to 190; martensite with different crystallographic orientations is set to an integer from 1 to 8. (2) Cell state variable: 1 represents pearlite (P); 2 represents ferrite (*α*); 3 represents austenite (*γ*); 4 represents interface between pearlite and ferrite (*α*–P interface); and 5 represents the interface between ferrite and austenite (*α*–*γ* interface). (3) Carbon concentration variables: carbon concentration of each cell at different times.

### 3.2. Kinetic Model and Austenite Transformation Rules

#### 3.2.1. Nucleation of Austenite

During the grind-hardening process, the austenite nucleus starts to be generated both inside the pearlite and at the *α*–P interface as soon as the temperature is higher than Ac1. This process does not end until all pearlite transforms into austenite. In the CA simulation, the probability of the austenite nucleation is always applied to calculate the number of nuclei. It is well known that pearlite is a composite of ferrite and cementite. Due to the difference in carbon concentration between ferrite and cementite, the austenite nucleation inside pearlite mainly appears at the interface between ferrite and cementite. However, for facilitating the calculation, it is assumed that pearlite is a single phase during CA simulation, and the probability of austenite nucleation inside the pearlite is seen as the same. Considering the difference in free energy, the probability of austenite nucleation at the *α*–P interface is approximately seven times more than that of pearlite [22].

The probability of austenite nucleation is given by

(7) $$P_{n}=\dot{n}\times \Delta t\times S,$$

where *S* is the area of each cell, and $S=L_{0}^{2}$. Δ*t* is time interval in CA simulation; and $\dot{n}$ is the austenite nucleation rate, which is given by

(8) $$\dot{n}=\alpha \times \varphi \times N_{d}\times f_{P}\times A,$$

where *α* is a coefficient, whose value is either 0.4 or 0.45; *φ* is the heating rate; *N*_d_ is the nucleation density; *f*_p_ is the pearlite fraction in the calculated domain; and *A* is the area of the calculated domain or subdomain.

In the simulation of austenite nucleation, a random number *rand*_1_ is assigned to each pearlite and the *α*–P interface cell in the CA lattice corresponds to a time interval. Then, *rand*_1_ is compared with *P*_n_. If *rand*_1_ ≤ *P*_n_, then the cell transforms into an austenite nucleus. A number from 11 to 190 is randomly assigned to the cell according to its crystallographic orientation. Based on the principle of large-angle interface nucleation, if the difference in orientation between two adjacent cells is less than 15, then the orientation of the cell will be renewed. Otherwise, the cell state does not change when *rand*_1_ > *P*_n_.

#### 3.2.2. Growth of Austenite

Since the grinding temperature is continuously increasing, the austenite nuclei begin to grow up once they are formed. For 1045 steel, the growth of austenite includes two processes, namely the dissolution of pearlite and the transformation from ferrite into austenite. For the dissolution of pearlite, the austenite nucleus forms the P–*γ* interface with the surrounding pearlite after it is formed inside the pearlite. With the rapid increase in temperature, the P–*γ* interface migrates towards the pearlite in the action of the transformation driving force, which finally makes all the pearlite transform into austenite. The driving force for the transformation from pearlite into austenite comes from the change in free energy and interfacial energy, and the energy barrier is considered to affect the free energy changing from one state to another. On this basis, the probability of transformation from pearlite into austenite can be described as a function related to the energy barrier, namely [23]:

(9) $$P_{P-\gamma }=\exp\left(\frac{-\Delta G^{t}}{RT}\right),$$

where Δ*G^t^* is the energy barrier; *R* is the gas constant; and *T* is absolute temperature.

In the CA simulation of pearlite dissolution, a random number *rand*_2_ is assigned to all the cells in turn at each time interval. Since the Moore neighbor is adopted in the simulation, eight neighbor cells around each pearlite cell were selected for further discussion. If austenite cells appear at neighboring cells, *rand*_2_ is compared with *P*_P–*γ*_ so as to determine whether austenitization appears on the pearlite cell. Once austenitization occurs, the cell state is modified from pearlite to austenite, and its crystallographic orientation is changed to be the same as that of the austenite cell which induces the transformation of pearlite.

Compared with the austenitization from pearlite, the transformation from ferrite to austenite is more complicated. Due to the low carbon concentration of ferrite, the transformation is affected by both carbon diffusion and *α*–*γ* interface migration. In fact, the austenite nucleation of the initial microstructure occurs together with carbon diffusion, whereas the carbon diffusion distance in pearlite is so short that the effect of carbon diffusion is always ignored during transformation. In other words, pearlite is recognized to be directly transformed into austenite. As for austenitization from ferrite, there is a large gradient for carbon concentration between ferrite and austenite. As the temperature rises, the carbon atoms in the austenite gradually diffuse to the ferrite when the austenite nuclei are formed in the *α*–P interface cell. The diffusion of carbon atoms makes the *α*–*γ* interface move in the direction of ferrite and finally achieve the transformation from ferrite into austenite. The carbon diffusion process will not stop until all ferrite cells transform into austenite. The governing equation of carbon diffusion among the microstructure with the influence of temperature is given by [24]

(10) $$\frac{\partial C_{E}}{\partial t}=D_{E}\left[\frac{\partial^{2}C_{E}}{\partial x^{2}}+\frac{\partial^{2}C_{E}}{\partial y^{2}}\right],$$

where *C*_E_ is equivalent concentration; *D*_E_ is diffusion coefficient in the *α*–*γ* interface cell. *C*_E_ and *D*_E_ are given by

(11) $$C_{E}=C_{\gamma }f_{\gamma }+C_{\alpha }\left(1-f_{\gamma }\right)=C_{\gamma }\left(f_{\gamma }+k\left(1-f_{\gamma }\right)\right),$$

(12) $$D_{E}=D_{\gamma }f_{\gamma }+kD_{\alpha }\left(1-f_{\gamma }\right),$$

where *C_α_* and *C_γ_* represent the carbon concentrations in the *α* and *γ* phases, respectively; *D_α_* and *D_γ_* are the carbon diffusion coefficients in both ferrite and austenite; *k* is a partition coefficient whose value is 0.02549.

The speed of *α*–*γ* interface migration during the transformation from ferrite to austenite is given by [25]

(13) $$v_{\alpha -\gamma }=M_{\alpha -\gamma }F,$$

where *M_α_*_–_*_γ_* is the mobility of the *α*–*γ* interface; *F* is the chemical driving force for *α*–*γ* interface migration.

*M_α_*_–_*_γ_* is given by

(14) $$M_{\alpha -\gamma }=M_{\alpha -\gamma }^{0}\exp\left(-\frac{Q_{\alpha -\gamma }}{RT}\right),$$

where $M_{\alpha -\gamma }^{0}$ is the pre-exponential factor; and *Q_α_*_–*γ*_ is the activation energy for interface diffusion.

If the mechanical driving force is ignored, *F* is given by [26]

(15) $$F=\left(1-X^{\alpha }\right)\left(\mu_{\mathrm{Fe}}^{\alpha }-\mu_{\mathrm{Fe}}^{\gamma }\right),$$

where $X^{\alpha }$ is the mole fraction of carbon atoms at the ferrite interface whose value is usually in the range of 0.01–0.015; $\mu_{\mathrm{Fe}}^{\alpha }$ and $\mu_{\mathrm{Fe}}^{\gamma }$ are the chemical potentials of iron atoms at the interface of both ferrite and austenite, respectively.

Since the value of $X^{\alpha }$ is close to 0, Equation (15) is simplified as

(16) $$F=\mu_{\mathrm{Fe}}^{\alpha }-\mu_{\mathrm{Fe}}^{\gamma },$$

Combining Equations (13)*–*(16), the migrating distance from the *α*–*γ* interface cell to ferrite within time Δ*t* is given by

(17) $$l_{\alpha -\gamma }=\int_{t_{0}}^{t_{0}+\Delta t}v_{\alpha -\gamma }dt,$$

In the CA simulation of the transformation from ferrite into austenite, *l_α_*_–*γ*_ is compared with the cell length. If *l_α_*_–*γ*_ ≥ *L*_0_, it can be considered that the *α*–*γ* interface cell transforms into austenite, and its crystallographic orientation is the same as the orientation of the austenite cell that induces the ferrite transformation. Meanwhile, the cells adjacent to the transformed austenite cell are set as the *α*–*γ* interface cell.

#### 3.2.3. Coarsening of Austenite

As the austenite grains continue to grow, austenite grains with different orientations may come into contact with each other. Since the *γ*–*γ* interface is rarely in equilibrium, larger grains grow at the expense of smaller ones due to the driving force of interface migration, which is regarded as the coarsening of austenite grains. When the *γ*–*γ* interface gradually goes into a straight line and the angle between the adjacent lines is close to 120°, the austenite grains reach a stable state.

During the coarsening process, the migrating speed of the *γ*–*γ* interface is given by [27,28]

*v_γ–γ_* = *M_γ–γ_P_c_*, (18)

where *M_γ_*_–*γ*_ is the *γ*–*γ* interfacial mobility; *P*_c_ is driving force for *γ*–*γ* interface migration.

*M_γ_*_–_*_γ_* is given by

(19) $$M_{\gamma -\gamma }=M_{\gamma -\gamma }^{0}\exp\left(-\frac{E_{\gamma -\gamma }}{RT}\right),$$

where $M_{\gamma -\gamma }^{0}$ is the pre-exponential factor; $E_{\gamma -\gamma }$ is the activation energy for interface migration.

*P*_c_ is given by

(20) $$P_{c}=\gamma_{i}\kappa ,$$

where *γ_i_* is the interfacial energy for austenite grain; *κ* is the curvature of the austenite interface.

### 3.3. Kinetic Model of Martensite Transformation

It can be seen from Figure 4 that the grinding temperature rises to the peak and then rapidly falls with a large cooling rate during the grind-hardening process. Based on the theory of phase transformation, the austenite on the workpiece surface transforms into martensite and finally forms a martensite lath with the driving force effect when the grinding temperature is lower than Ms. In martensitization process, the formation of martensite lath mainly contains two processes—namely martensite nucleation and grain growth. The nucleation of martensite lath is concentrated at the interface of the austenite grain. The nucleation rate of the martensite lath is given by [14]

(21) $$N_{m}=n_{i}\upsilon \exp\left(\frac{-\Delta G_{a}}{kT}\right),$$

where *n_i_* is the assumed potential number of nuclei; *υ* is the lattice vibration frequency; Δ*G_a_* is the activation energy of nucleation; and *k* is Boltzmann constant.

Since the martensite in the hardened layer is formed during the rapid cooling, the amount of martensite only depends on the cooling temperature and has nothing to do with time. The martensite fraction *V* at different cooling temperatures can be calculated by [29]

(22) $$V=1-\exp\left(-\Gamma \frac{T^{*}-T_{q}}{T_{q}}\right),$$

where Γ is a dimensionless parameter related to factors such as the workpiece material and martensite nucleation number; T* is defined as the maximum temperature at which the martensite nucleus becomes viable and whose value is close to Ms; and *T*_q_ is the cooling temperature.

In the CA simulation of martensite transformation, the martensite nucleus is distributed at positions both on the austenite interface and adjacent to the existing martensite lath. Once the martensite nucleus is formed, it grows in eight directions at an angle of 45° between each direction [30]. In the growth process, the martensite lath cannot go across an austenite interface or penetrate each other.

### 3.4. Simulation and Discuss

**Figure 7:**
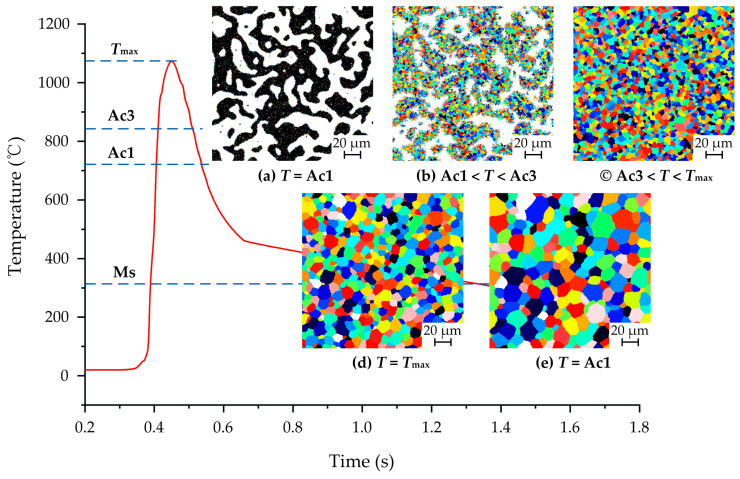
Austenite transformation on workpiece surface at different heating temperatures: (**a**) *T* = Ac1; (**b**) Ac1 < *T* < Ac3; (**c**) Ac3 < *T* < *T*_max_; (**d**) *T* = *T*_max_; (**e**) *T* = Ac1.

In CA simulation, it is necessary to record the grinding temperature of each cell in the simulated area at different times since the microstructure transformation is greatly affected by grinding heat. The grinding temperatures varying at different times are obtained by FE simulation (as shown in Figure 4). The austenitization process of the initial microstructure on the surface during grind-hardening is shown in Figure 7. It can be seen from Figure 7a that when the grinding temperature reaches Ac1, the austenite nuclei with different orientations gradually appear both inside the pearlite and at the *α*–P interface. As the temperature gradually rises, the pearlite completely transforms into austenite relatively quickly. When the pearlite is completely transformed into austenite, the ferrite transforms into austenite because of the interfacial driving force caused by the carbon diffusion between the austenite and ferrite as well as the temperature variation. The ferrite will totally transform into austenite as soon as the temperature rises above Ac3, as shown in Figure 7b,c. Then, the coarsening process starts among the austenite grains with different orientations as the temperature varies and does not stop until the grinding temperature drops below Ac1. At this moment, the austenite grain interfaces appear to be straight with an angle between each interface which tends towards 120°, as shown in Figure 7d,e.

**Figure 8:**
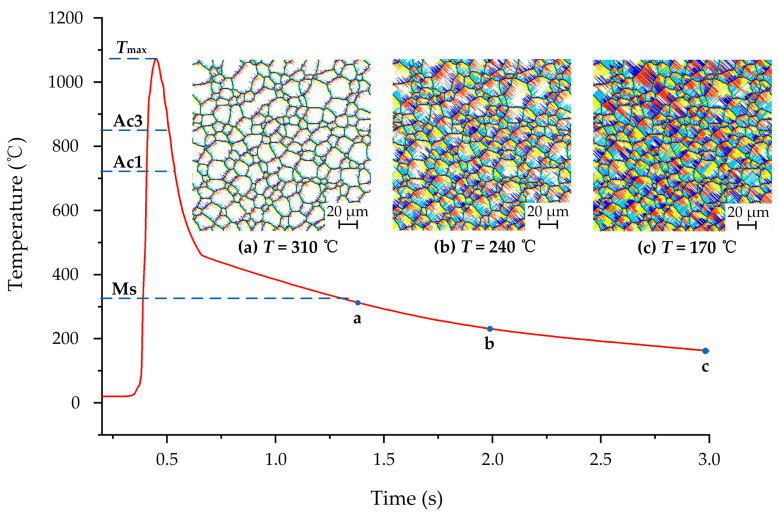
Martensite transformation on workpiece surface at different cooling temperatures: (**a**) *T* = 310 °C; (**b**) *T* = 240 °C; (**c**) *T* = 170 °C.

The transformation process from austenite into martensite on the workpiece surface is shown in Figure 8. In the figure, the lines with different colors represent martensite with different crystallographic orientations. It can be seen from Figure 8a that when the grinding temperature is cooled to Ms, the martensite nucleus is generated at the austenite interface. In addition, martensite with different orientations starts to grow in different directions. Figure 8b,c shows the distribution of martensite on the surface at different cooling temperatures. As the grinding temperature decreases, the martensite nucleus continues to generate and rapidly grows at both the austenite interface and adjacent to the existing martensite lath. It makes the martensite fraction on the surface gradually increase, which eventually forms a hardened layer dominated by martensite. The simulation shows that the final martensite fraction in the hardened layer with *a*_e_ = 350 μm, *v*_w_ = 9 m/min and *v*_s_ = 26.4 m/s is 79%.

## 4. Hardness Prediction for Hardened Layer

### 4.1. Hardness Modeling

The hardness of the hardened layer is predicted by the hardness superposition principle. From the principle, the hardness of the hardened layer is related to the hardness and action of each microstructure on the workpiece surface [16]. The Vickers hardness of the hardened layer is given by

(23) $$\mathrm{Hv}=\xi^{\left(M\right)}{\mathrm{Hv}}^{\left(M\right)}+\xi^{\left(B\right)}{\mathrm{Hv}}^{\left(B\right)}+\left(\xi^{\left(F\right)}+\xi^{\left(P\right)}\right){\mathrm{Hv}}^{\left(F+P\right)},$$

where *ξ*^(M)^, *ξ*^(B)^, *ξ*^(F)^, *ξ*^(P)^ are the fractions of martensite, bainite, ferrite, and pearlite, respectively; Hv^(M)^, Hv^(B)^, Hv^(F+P)^ are the hardness of martensite, bainite, ferrite, and pearlite, respectively. The hardness of the microstructure is related to the chemical composition of the workpiece material and the cooling rate, namely:

(24) $${\mathrm{Hv}}^{\left(M\right)}=127+949w_{C}+27w_{\mathrm{Si}}+11w_{\mathrm{Mn}}+8w_{\mathrm{Ni}}+16w_{\mathrm{Cr}}+21\log v_{r},$$

(25) $$\begin{array}{l} {\mathrm{Hv}}^{\left(B\right)}=-323+185w_{C}+330w_{\mathrm{Si}}+153w_{\mathrm{Mn}}+65w_{\mathrm{Ni}}+144w_{\mathrm{Cr}}+191w_{\mathrm{Mo}}\\ \;\;\;+\left(189+53w_{C}-55w_{\mathrm{Si}}-22w_{\mathrm{Mn}}-10w_{\mathrm{Ni}}-20w_{\mathrm{Cr}}-33w_{\mathrm{Mo}}\right)\log v_{r} \end{array}$$

(26) $$\begin{array}{l} {\mathrm{Hv}}^{\left(F+P\right)}=42+223w_{C}+53w_{\mathrm{Si}}+30w_{\mathrm{Mn}}+12.6w_{\mathrm{Ni}}+7w_{\mathrm{Cr}}+19w_{\mathrm{Mo}}\\ \;\;\;\;+\left(10-19w_{\mathrm{Si}}+4w_{\mathrm{Ni}}+8w_{\mathrm{Cr}}+130w_{V}\right)\log v_{r} \end{array}$$

where *v*_r_ is the cooling rate of the workpiece at 700 °C and *v*_r_ = (800 − 500)/Δ*t*_c_; Δ*t*_c_ is time interval when the workpiece is cooled from 800 to 500 °C.

### 4.2. Hardness Prediction

**Figure 9:**
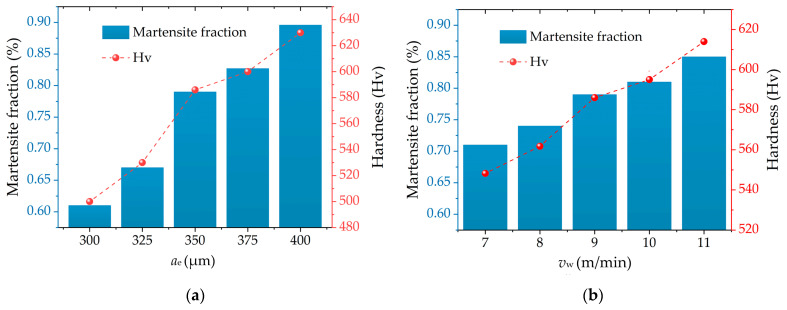
Prediction of martensite fraction and hardness at different grinding parameters: (**a**) *v*_w_ = 9 m/min, *v*_s_ = 26.4 m/s; (**b**) *a*_e_ = 350 μm, *v*_s_ = 26.4 m/s.

It can be seen from the start that the generation of the hardened layer can significantly improve the surface hardness of the workpiece, which is mainly attributed to the martensite transformation. Therefore, it is possible to determine the martensite fraction in the hardened layer—first by means of CA simulation. Then, the hardness superposition principle is applied to predict the hardness of hardened layer with different grinding parameters. Figure 9 shows the martensite fraction of the hardened layer and its hardness predictive value at different grinding parameters. It can be seen from the figure that the martensite fraction and hardness rise with the increase in *a*_e_ and *v*_w_. The reason is that as *a*_e_ and *v*_w_ increase, the grinding force and temperature generated during the grind-hardening process are greatly increased. Simultaneously, the cooling rate of the workpiece surface rises as well. This does not only cause the increase in the fraction and martensite hardness on the surface, but also eventually enhances the hardness of the hardened layer. In addition, *v*_w_ has a smaller impact on the hardness of the hardened layer than *a*_e_, which is mainly because *a*_e_ has a greater impact on the grinding temperature than *v*_w_.

## 5. Grind-Hardening Experiment

The hardness prediction of the hardened layer was verified by grind-hardening experiment. The experimental conditions such as equipment, workpieces, and grinding parameters used in the experiment are shown in Table 3.

**Figure 10:**
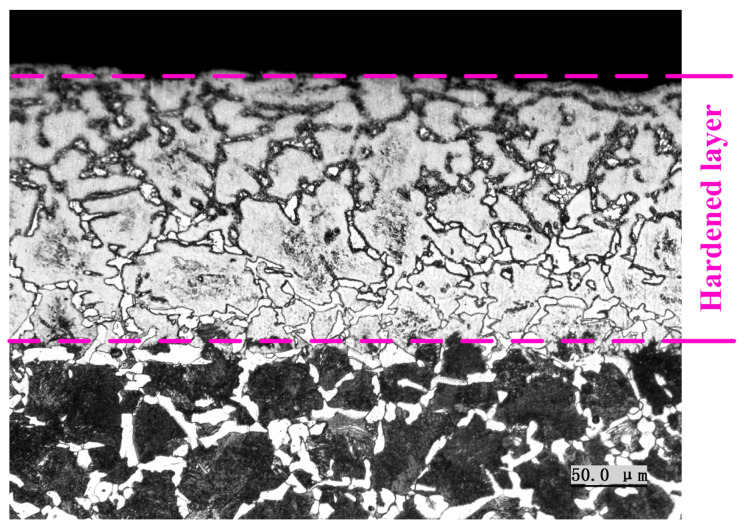
The microstructure distribution after grind-hardening (*a*_e_ = 350 μm, *v*_s_ = 9 m/min, *v*_s_ = 26.4 m/s).

A metallurgical microscope (Olympus Corporation, Tokyo, Japan) was applied to observe the microstructure of the workpiece surface after grind-hardening, as shown in Figure 10. From the figure, the initial microstructure of the workpiece surface appears as a martensite transformation with the influence of grinding heat, which thereby forms a hardened layer with a certain depth. The microstructure of the hardened layer is mainly composed of martensite lath, a small amount of retained austenite, and undissolved carbides. Its depth is mainly related to factors such as the workpiece material, grinding temperature, and cooling rate [31]. It should be noted that there is no bainite in the hardened layer. This is mainly due to the high carbon content of the undercooled austenite on the workpiece surface during the cooling process, which significantly prolongs the time of the bainite transformation. When the undercooled austenite is continuously cooled through the bainite transformation temperature zone, the bainite transformation process still cannot be completed, which causes the austenite to directly form martensite without undergoing bainite transformation.

**Figure 11:**
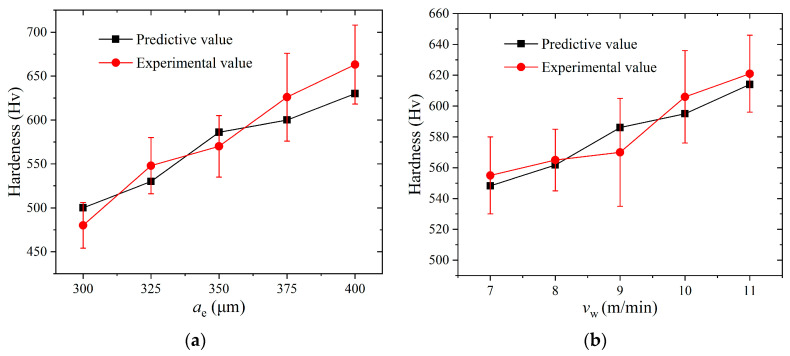
Comparison of the predictive value and experimental value of the surface hardness: (**a**) *v*_w_ = 9 m/min, *v*_s_ = 26.4 m/s; (**b**) *a*_e_ = 350 μm, *v*_s_ = 26.4 m/s.

The Vickers hardness tester was adopted to measure the hardness of the hardened layer at different grinding depths. Figure 11 illustrates the comparison between the experimental value and predictive value. It can be seen from Figure 11 that the hardness at different grinding depths is much higher than the initial hardness of the material (180 Hv). Furthermore, the experimental value and predictive value of the hardness appear with the same tendency as the grinding depth variations. Our comparison finds that the predictive value is much closer to the experimental value. The error between the two values is within an allowable range. The main reasons for the error are as follows. Firstly, there are instantaneous fluctuations in the grinding force when the wheel grinds on the workpiece due to the blockage of the wheel, and the fracture of abrasive grains and the chattering of the machine tool. It will increase the error of the grinding force between the theoretical value and the experimental value, which will significantly affect the predictive accuracy of the hardness. Furthermore, it is known that the microstructure of the 1045 steel is mainly composed of ferrite and pearlite. Whereas the grain size and distribution of the workpiece surface applied in the experiment are uncertain, these will elucidate the difference in the initial microstructure of the workpiece in the CA simulation. This is also an important cause of error. From the above analysis, the combination of FE and CA is an effective and reliable approach for predicting the hardness of a hardened layer.

## 6. Conclusions

In this study, the grinding temperature variation and the microstructure transformation of the surface for the 1045 steel were simulated by an integrated approach of FE and CA. Moreover, the hardness of the hardened layer with different parameters was predicted. The predicted results were also verified by grind-hardening experiment.

(1) The FE simulation of the distribution and variation of the surface temperature of 1045 steel workpiece was carried out on the basis of the grind-hardening heat source model. This study found that the model was helpful in simulating the variation of the grinding temperature when the wheel was grinding the workpiece. When the grinding parameters were taken to be *a*_e_ = 350 μm, *v*_w_ = 9 m/min, *v*_s_ = 26.4 m/s, the maximum temperature was up to 1097.6 °C. The surface temperature presents a trend of first increasing and then decreasing. Among others, the highest temperature is higher than Ac3, and the lowest temperature is lower than Ms. From this point of view, it can be considered that the workpiece surface has formed a hardened layer mainly composed of martensite. This conclusion is consistent with the experimental results.

(2) Based on the FE analysis of the grinding temperature field, the transformation process from the initial microstructure to austenite first and then to martensite with the influence of grinding heat is simulated by CA approach. Studies have shown that the integration of the FE and CA approach can simulate the transformation process very well, which is helpful to realize the accurate prediction of the hardness.

(3) The hardness of the hardened layer with different grinding parameters was predicted by martensite fraction calculation and the hardness superposition principle. The results show that the martensite fraction in the hardened layer and its hardness rose with the increase in *a*_e_ and *v*_w_, which were mainly attributed to the increase in grinding temperature and cooling rate as *a*_e_ and *v*_w_ grew. Finally, the predictive value of the hardness and its variation were proved to be reasonable and effective by grind-hardening experiment.

## Figures and Tables

**Table 1:** Chemical composition of 1045 steel.

Component	C	Si	Mn	Cr	Ni	Cu
Fraction (%)	0.42–0.5	0.17–0.37	0.5–0.8	≤0.25	≤0.3	≤0.25

**Table 2:** Material properties of 1045 steel.

**Temperature (°C)**	20	100	200	300	400	500	600	700	800	900	1000
**Density (kg/m^3^)**	7850	7830	7800	7770	7740	7700	7685	7672	7660	7651	7649
**Specific Heat (J/kg·°C)**	460	480	498	524	524	615	690	720	682	637	602
**Heat Conductivity (W/m·°C)**	49.77	46.76	43.24	40.29	37.87	35.96	33.18	30.52	27.96	25.92	24.02

**Table 3:** The experimental conditions.

Experimental Conditions		Parameters
Machine		BLOHM ORBIT 36CNC (Korber Schleifring, Shanghai, China)
Wheel	Size (outer diameter × width)	350 mm × 40 mm
	Granularity	F46
	Abrasive material	White alumina
Workpiece	Material	1045 steel
	Size	90 mm × 9 mm × 14 mm
Cooling mode and grinding method		Dry grinding, up grinding
Metallographic observation equipment		OLYMPUS-GX71 (Olympus Corporation, Tokyo, Japan)
Vickers hardness test instrument		THV-5 (Beijing Time High Technology Ltd., Beijing, China)
Grinding parameters	Grinding depth *a*_e_ (μm)	300, 325, 350, 375, 400
	Feeding speed *v*_w_ (m/min)	7, 8, 9, 10, 11
	Wheel speed *v*_s_ (m/s)	26.4

## Data Availability

The data presented in this study are available on request from the corresponding authors.
